# Supplementary material for: The genome sequence of the Large Red Damselfly Pyrrhosoma nymphula (Sulzer, 1776)
Source: Wellcome Open Res. 2024 Oct 15;9:367. Originally published 2024 Jul 10. [Version 2] doi: 10.12688/wellcomeopenres.22586.2 (PMC11342034; doi:10.12688/wellcomeopenres.22586.2)
Supplement: Peer review history

# Reviewer report: Reviewer response for version 1

**DOI:** 10.21956/wellcomeopenres.24884.r92424

**Published:** 2024-08-22

**Authors:** Gurinder Kaur Walia

**Affiliations:** 1 Punjabi University, Patiala, Punjab, India

**Strengths**

 The paper focuses on the technical aspects of the genome assembly, with relatively little discussion on the biological implications of the genomic data. There is limited discussion on how this genome sequence compares to other damselflies. Comparative genomics could provide more context on the uniqueness and conservation of genomic features.

Are sufficient details of methods and materials provided to allow replication by others?

Yes

Is the rationale for creating the dataset(s) clearly described?

Yes

Are the datasets clearly presented in a useable and accessible format?

Yes

Are the protocols appropriate and is the work technically sound?

Yes

Reviewer Expertise:

Molecular cytogenetics

I confirm that I have read this submission and believe that I have an appropriate level of expertise to confirm that it is of an acceptable scientific standard.

# Reviewer report: Reviewer response for version 1

**DOI:** 10.21956/wellcomeopenres.24884.r92432

**Published:** 2024-08-22

**Authors:** Yesenia Vega-Sánchez

**Affiliations:** 1 Instituto de Investigaciones en Ecosistemas y Sustentabilidad, Universidad Nacional Autónoma de México (UNAM), Morelia, Mexico

The Data Note describes a chromosome-level genome assembly of *Pyrrhosoma nymphula*, an insect species of the Order Odonata.

 In general, the manuscript is well developed; except for some paragraphs that seem redundant, for example the first and last paragraphs of the Background. The methods are concise and sufficient for the repeatability of the study. The results are clear, however, in my opinion, there are some images that are not necessary (e.g. Fig. 3 and Fig. 4). I have no additional comments.

Are sufficient details of methods and materials provided to allow replication by others?

Yes

Is the rationale for creating the dataset(s) clearly described?

Yes

Are the datasets clearly presented in a useable and accessible format?

Yes

Are the protocols appropriate and is the work technically sound?

Yes

Reviewer Expertise:

Population genomics, evolution, insects systematics.

I confirm that I have read this submission and believe that I have an appropriate level of expertise to confirm that it is of an acceptable scientific standard.

# Reviewer report: Reviewer response for version 1

**DOI:** 10.21956/wellcomeopenres.24884.r92425

**Published:** 2024-08-22

**Authors:** Beatriz Willink

**Affiliations:** 1 Department of Zoology, Stockholm University, Stockholm, Sweden; 2 National University of Singapore, Singapore, Singapore

This data note describes an assembly for the Large Red Damselfly *Pyrrhosoma nymphula. *Damselflies are ancient insects with striking ecological, behavioural, and morphological diversity, but with relatively limited genomic resources to investigate the molecular basis, evolution, and threats to this diversity. I thus think that this work provides an important resource for future research. The data is of high quality and the note is generally well written. I have a few suggestions to improve clarity and provide biological context to the manuscript.

 1. The Background section briefly mentions the occurrence of sexual dimorphism in colour and female-limited colour morphs in *P. nymphula*:

 “Black markings on the abdomen occur in three forms in the female and are less numerous in the male”.

 I think this brief mention could be expanded to highlight one of the potential uses of this assembly. *P. nymphula *belongs to Coenagrionidae, a damselfly family in which females often display colour polymorphisms, but in which males are typically monomorphic (see for example Fincke et al. 2005). As the authors mention, there are three female morphs in *P. nymphula*, which vary in the extent of black abdomen markings and thus in the extent of their resemblance to males (Askew 2004).

 This genome assembly creates the opportunity to investigate the genetic basis of this sex-specific colour variation. There have been a few recent studies looking into the genetics of female-limited polymorphisms in coenagrionid damselflies (Takahashi et al. 2019, Willink et al. 2024), but I think *P. nymphula *is really interesting, because female morphs in this species show a gradient of phenotypic differentiation from males. One morph ( *fulvipes*) has a mostly red abdomen, similar to males. Another morph ( *melanotum*) has a mostly black abdomen, and the third morph ( *intermedia* or *typica*) has some red and some black markings. This assembly provides a great resource to investigate the genetics of this polymorphism, perhaps in the larger context of the evolution of sexual dimorphism.

 2. Another potential application for this assembly could be for biogeographic and speciation research. I’m not so familiar with this literature, but I understand there are two lineages that diverged from the more widespread *P. nymphula *lineage, one in Northern Africa, and one in costal Greece. The lineage in costal Greece, currently considered a separate species ( *P. elisabethae*), is critically endangered (see IUCN redlist). There is some older and more recent research on this topic that might be worth revising (e.g. Kalkman and Lopau 2006, Guan et al. 2013, Simonsen et al. 2024). I imagine that this assembly can help shed light into the interesting biogeographic history and evolutionary relationships within this clade.

 3. I understand this is the most common sex determination system in Odonata, but I wonder how the authors determined that their male sample is XO.

 4. Fig 3: this is explained in the text, but the x and y axes should also be described in the figure legend as well. “gc” and “ERR6688447_cov” are not intuitive axes titles.

 5. Perhaps the legend of Figure 4 use "scaffolds" instead of "sequence". Otherwise, it’s ambiguous whether “sequence” refers to raw reads or assembled scaffolds.

 6. Fig. 4: Why is the background different in chromosome 6? Are there fewer HiC contacts? Or may be it just looks so against the white background. I think clarifying this is required to make the article scientifically sound.

 7. I found that the “Evaluation of final assembly” section was difficult to follow. I think that revising this section is required to make the article scientifically sound. Specifically:

 “The sanger-tol/genomenote pipeline transforms the Hi-C alignments into a contact map with BEDTools ( Quinlan & Hall, 2010) and the Cooler tool suite ( Abdennur & Mirny, 2020), which is then visualised with HiGlass ( Kerpedjiev *et al.*, 2018).”

 This long sentence would be easier to read if split into two. One suggestion, assuming the meaning is preserved, could be:

 “The sanger-tol/genomenote pipeline transforms the Hi-C alignments into a contact map with BEDTools ( Quinlan & Hall, 2010) and with the Cooler tool suite ( Abdennur & Mirny, 2020). The contact map was then visualised with HiGlass ( Kerpedjiev *et al.*, 2018).”

 In the following sentence “It also provides statistics about the assembly...”, clarify what you are referring to with “It”. For example, “The sanger-tol/genomenote also provides statistics about the assembly...” or “HiGlass also provides statistics about the assembly...”, whichever is correct.

 Also in this sentence, I think the citation should be placed after “report”.

 The next paragraph describes the sanger-tol/blobtoolkit, but it is unclear to me what are the results of this evaluation step. The paragraph mentions coverage tracks for regions of a fixed size, BUSCO analyses for domain-level lineages, alignments to the Uniprot Reference Proteomes and to the NT database, which are all combined into a “blobdir for visualisation”. Should this visualization be on the paper or available through a link?

 The snail plot on Fig. 2 shows other, perhaps more basic assembly statistics. Considering that this sanger-tol/blobtoolkit pipeline is relatively new, it might be useful to mention in not so technical language how the pipeline contributes to the evaluation of the assembly, in addition of presenting its results.

 8. There is an extra space before “.” at the end of the first paragraph, in the “Background” section.

Are sufficient details of methods and materials provided to allow replication by others?

Yes

Is the rationale for creating the dataset(s) clearly described?

Partly

Are the datasets clearly presented in a useable and accessible format?

Partly

Are the protocols appropriate and is the work technically sound?

Yes

Reviewer Expertise:

Evolutionary biology

I confirm that I have read this submission and believe that I have an appropriate level of expertise to confirm that it is of an acceptable scientific standard, however I have significant reservations, as outlined above.

## References

[1] : Preliminary Material.2004; 10.1163/9789004474383_001 1-5 10.1163/9789004474383_001

[2] : Pyrrhosoma and its relatives: a phylogenetic study (Odonata: Zygoptera). *International Journal of Odonatology* .2013;16(3) : 10.1080/13887890.2013.821358 247-257 10.1080/13887890.2013.821358

[3] : The evolution and frequency of female color morphs in Holarctic Odonata: why are male-like females typically the minority?. *International Journal of Odonatology* .2005;8(2) : 10.1080/13887890.2005.9748252 183-212 10.1080/13887890.2005.9748252

[4] : Identification ofPyrrhosoma elisabethae with notes on its distribution and habitat (Odonata: Coenagrionidae). *International Journal of Odonatology* .2006;9(2) : 10.1080/13887890.2006.9748276 175-184 10.1080/13887890.2006.9748276

[5] : Increased geographic sampling suggests incomplete lineage sorting and recent introgression between Pyrrhosoma nymphula (Sulzer, 1776) and P. elisabethae Schmidt, 1948 in the Western Palearctic. *International Journal of Odonatology* .2024;27: 10.48156/1388.2024.1917265 37-46 10.48156/1388.2024.1917265

[6] : Candidate genes associated with color morphs of female-limited polymorphisms of the damselfly Ischnura senegalensis. *Heredity (Edinb)* .2019;122(1) : 10.1038/s41437-018-0076-z 81-92 10.1038/s41437-018-0076-z 29713090 PMC6288084

[7] : The genomics and evolution of inter-sexual mimicry and female-limited polymorphisms in damselflies. *Nat Ecol Evol* .2024;8(1) : 10.1038/s41559-023-02243-1 83-97 10.1038/s41559-023-02243-1 37932383 PMC10781644
